# Linking Environmental Forcing and Trophic Supply to Benthic Communities in the Vercelli Seamount Area (Tyrrhenian Sea)

**DOI:** 10.1371/journal.pone.0110880

**Published:** 2014-10-24

**Authors:** Anabella Covazzi Harriague, Giorgio Bavestrello, Marzia Bo, Mireno Borghini, Michela Castellano, Margherita Majorana, Francesco Massa, Alessandro Montella, Paolo Povero, Cristina Misic

**Affiliations:** 1 Dipartimento di Scienze della Terra, dell'Ambiente e della Vita - DiSTAV – University of Genoa, Italy; 2 CNR-ISMAR, Institute of Marine Sciences, National Research Council, Section of La Spezia, Pozzuolo di Lerici, Italy; The Evergreen State College, United States of America

## Abstract

Seamounts and their influence on the surrounding environment are currently being extensively debated but, surprisingly, scant information is available for the Mediterranean area. Furthermore, although the deep Tyrrhenian Sea is characterised by a complex bottom morphology and peculiar hydrodynamic features, which would suggest a variable influence on the benthic domain, few studies have been carried out there, especially for soft-bottom macrofaunal assemblages. In order to fill this gap, the structure of the meio-and macrofaunal assemblages of the Vercelli Seamount and the surrounding deep area (northern Tyrrhenian Sea – western Mediterranean) were studied in relation to environmental features. Sediment was collected with a box-corer from the seamount summit and flanks and at two far-field sites in spring 2009, in order to analyse the metazoan communities, the sediment texture and the sedimentary organic matter. At the summit station, the heterogeneity of the habitat, the shallowness of the site and the higher trophic supply (water column phytopigments and macroalgal detritus, for instance) supported a very rich macrofaunal community, with high abundance, biomass and diversity. In fact, its trophic features resembled those observed in coastal environments next to seagrass meadows. At the flank and far-field stations, sediment heterogeneity and depth especially influenced the meiofaunal distribution. From a trophic point of view, the low content of the valuable sedimentary proteins that was found confirmed the general oligotrophy of the Tyrrhenian Sea, and exerted a limiting influence on the abundance and biomass of the assemblages. In this scenario, the rather refractory sedimentary carbohydrates became a food source for metazoans, which increased their abundance and biomass at the stations where the hydrolytic-enzyme-mediated turnover of carbohydrates was faster, highlighting high lability.

## Introduction

The deep-sea communities of the Mediterranean Sea (namely those living below a 200 m depth) have been investigated rather intensively, but these studies have typically been characterised by a limited spatial or temporal scale of investigation [1], [2], [3], [4], [5], [6]. Focusing on the deep Tyrrhenian Sea, very few papers have been published on microbial benthic communities [7] and meiofaunal communities [8] and studies on deep, soft-bottom macrofauna are lacking. This is surprising as the Tyrrhenian Sea hosts a number of morphological peculiarities (seep, vent, slope, and abyssal plain habitats, and seamounts etc.) that have led us to suppose that an interesting part of the Mediterranean's biodiversity could be hidden there.

The northwestern Tyrrhenian Sea is characterised by a complex hydrology [9], [10], which responds to the bottom morphology. A submerged ridge, called the Vercelli Seamount, with its main axis SW-NE, reaches the photic layer from the bathyal plain [11], [12]. Almost permanent frontal zones exist on the main Vercelli Seamount axis, modifying the pelagic-benthic coupling, while cyclonic and anticyclonic gyres move the water masses around it [13].

In this complex scenario, the forcings that usually shape benthic communities (depth, sediment texture, trophic supply) may change their roles suddenly. The presence of a Taylor column [14] and the impinging of water circulation on the seamount's flanks have been suggested as pivotal factors for the development of the benthic communities, for instance where the presence of a rather stable Taylor column isolates the summit and limits foraging of the down-current zones [15], [16]. Higher and lower current speeds have been invoked to explain community trophic differences on different flanks of the seamount [11]. In such systems a constant pelagic-benthic coupling, providing the aphotic sediment with food, is hardly possible, strongly influencing the benthic assemblages [14], [17], [18], [19]. All these variables and the variable slope of the flanks, that may exert a certain influence on the sediment texture, influence the characteristics of the benthic community, and have encouraged the hypothesis that seamounts and the surrounding area are peculiar hotspots ofmarine life [20], [21], [22].

This study aims to link the metazoan benthic community (meiofauna and macrofauna) to the environmental constraints, highlighting how the parameters considered (morphological as well as trophic) may have a role in community characterisation.

## Material and Methods

### Study area, sampling sites and sampling strategy

**Figure 1:**
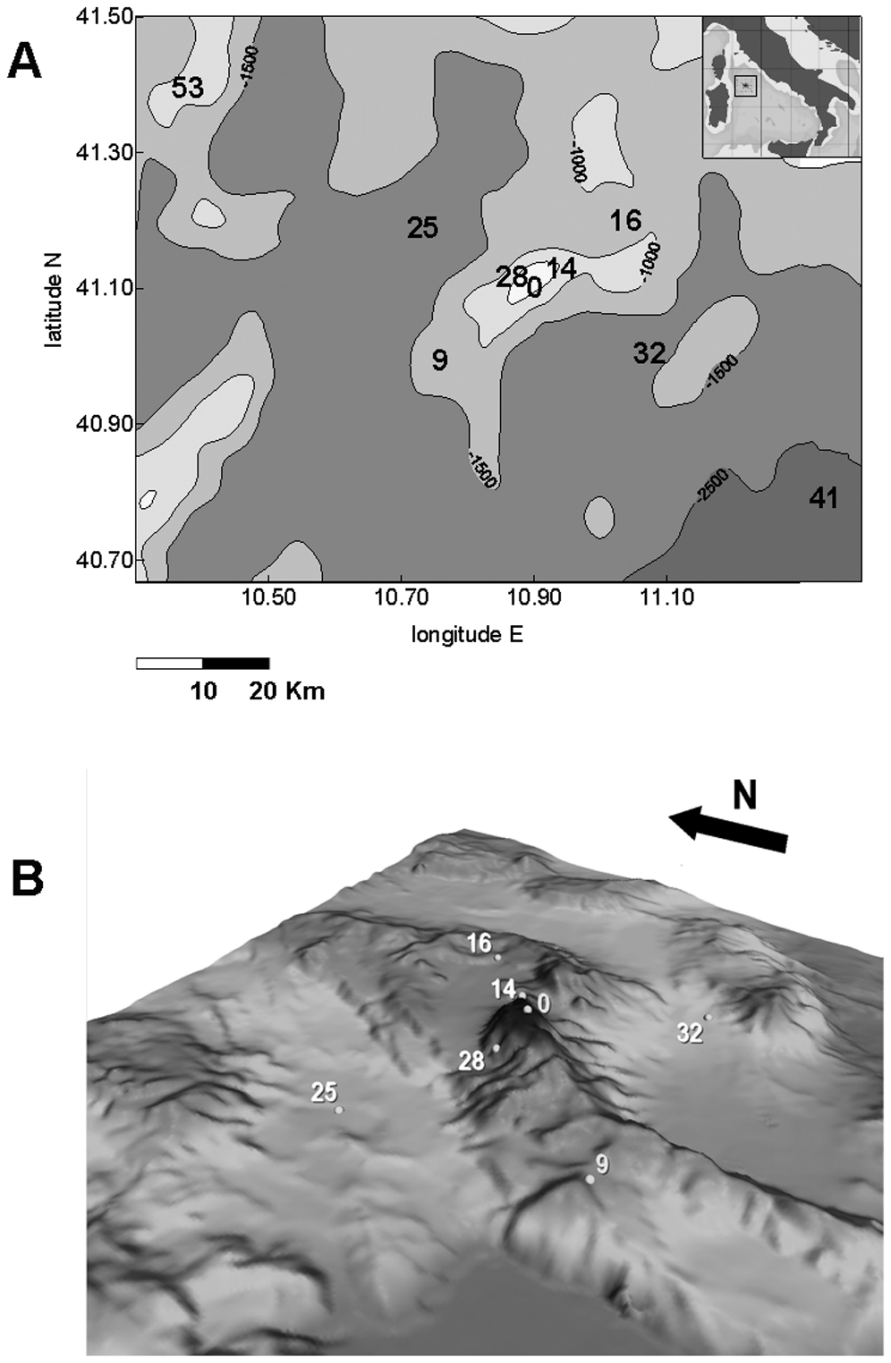
View of the Vercelli Seamount area (Tyrrhenian Sea). The bathymetric map (A) shows that the seamount has a SW-NE oriented axis and the summit (41°06′N/10°54′E) rises from the bathyal plain to a depth of 55 m. All the stations where sediment has been sampled are shown (see Table 1 for further details). The three-dimensional detail of the seamount (map B) is centred on the summit station 0 (at 115 m depth), the upper flank stations 14 and 28 (at 400 and 877 m depth), the medium-flank stations 9 and 16 (at 1232 and 1166 m depth), and the deeper-flank stations 25 and 32 (at 1833 and 1728 m depth).

The sampling area lies in the NW Tyrrhenian Sea (NW Mediterranean), near Sardinia (Fig. 1), and it is located within the following coordinates: 40°46′N/10°39′E, 40°47′N/11°34′E, 41°24′N/11°34′E and 41°24′N/10°38′E. It is centred on the Vercelli Seamount, an elongated, chain seamount whose axis is oriented SW-NE, and whose summit (41°06′N/10°54′E) rises from the bathyal plain to a depth of 55 m. The seamount summit covers an area of about 0.36 km^2^, characterised by alternating rocky and sandy surfaces of variable depths. About half of the area lies between 100 and 120 m, while only 15% is shallower than 80 m.

This area has been studied previously for its hydrodynamic features, within the framework of research on the Tyrrhenian Sea circulation and water mass fluxes. The previous studies showed the presence of a large cyclonic structure (the Bonifacio gyre) [23] that displayed permanent features, centred NW of the sampling area and crossing it in its W sector [9], [13]. Krivosheya [24] noted the presence of an anticyclonic companion of the Bonifacio gyre to the southeast. The Vercelli Seamount is placed within the transition area between the two gyres [9], whose boundaries are frontal zones [10]. In addition, Vetrano et al. [13]noted the presence of another mesoscale structure in the NE section of the sampling area, although less stable than the others. All these structures extend from the surface to considerable depths [13].

Sediment and water samples were collected from the R/V *Urania* during May 2009. During our field studies endangered or protected species were not involved and the permit for sampling activity was issued by the Italian Ministry of Defence (Military Navy) and the Italian Ministry of Communications. Nine stations were visited (Fig. 1). Station 28 was only sampled for organic matter (OM), and enzymatic and granulometric parameters due to the steepness of the seafloor, which prevented proper closure of the sampling device, and station 0 only for macrofauna due to the risk of damage to the sampling device on the irregular sandy-rocky bottom.

The stations (Table 1) were located on the summit (station 0) and around the seamount at different distances from the summit. Stations 14 and 28 were located on the upper-flank area, NE and NW of the summit respectively, where the flanks were rather steep. The other stations were located at different depths on the seamount's flanks, stations 9 and 16 at medium depths (1100–1200 m) and stations 25 and 32 at lower depths (1700–1800 m), close to the bathyal plain. Station 41 was located in the extreme SE sector of the study area and up-current from the seamount. Station 53 was, instead, placed in the extreme NW sector, influenced by the permanent Bonifacio gyre. Both stations were considered control sites, being about 50 km from the seamount summit, although having very different depths and features.

**Table 1:** Location of sampling stations with respect to the seamount morphology and to the general current flow and evaluated variables.

station	latitude N	longitude E	depth (m)	location	bottom inclination (°)	exposition (°N)	current from (a)	sampling parameters
0	41.108	10.907	115	summit	1,70	77	SE	Gr, Ma
9 *	40.993	10.759	1232	medium flank	8,49	128	SW	Gr, OM, E, Me, Ma
14	41.128	10.940	400	upper flank	14,61	20	SE	Gr, OM, E, Me, Ma
16 *	41.200	11.038	1166	medium flank	4,91	261	N-NW	Gr, OM, E, Me, Ma
25	41.188	10.733	1833	deeper flank	0,36	352	SE	Gr, OM, E, Me, Ma
28	41.117	10.867	877	upper flank	17,46	332	SE	Gr, OM, E
32 *	41.003	11.074	1728	deeper flank	2,84	287	SE	Gr, OM, E, Me, Ma
41 *	40.790	11.339	2646	far-field	5,23	302	SE	Gr, OM, E, Me, Ma
53 *	41.394	10.378	887	far-field	5,84	238	W-SW	Gr, OM, E, Me, Ma

Asterisks denote those stations whose bottom inclination doesn't follow the seamount flank general pendency.

Gr: granulometry, OM: organic matter, E: enzymatic activity, Me: meiofauna, Ma: macrofauna.

(a): general current flows as reported in Vetrano et al. [13]

Undisturbed sediment cores were collected with independent deployments using a box-corer with a 29 cm internal diameter. Immediately after the arrival of the box-corer on board, the overlaying bottom water was gently removed without notably disturbing the sediment surface. For the granulometric, OM, enzymatic activity and meiofaunal analyses, the sediment in the box-corer was subsampled by pushing PVC cores (5 cm internal diameter) into it, in duplicate for each analysis except meiofaunal, which was collected in triplicate. Each core was then aseptically sliced (at 0–2 and 2–10 cm depths). Each sediment slice was frozen until analysis, except for the enzymatic activity determinations, that were performed immediately. For the macrofaunal analyses, 3 deployments were performed for each station and entirely sorted with a 500 µm mesh net. The limited sampling for meiofauna could have reduced the representativeness of the data. In particular, the rare species could have been underestimated, thus leading only to preliminary considerations on meiofaunal diversity.

In addition to the sediment sampling, we also performed surface-water layer sampling at 25 stations distributed throughout the sampling area. Four depths were sampled with Niskin bottles: surface, oxygen minimum (between 30 and 50 m), fluorescence maximum (between 55 and 75 m), depth of extinction of the fluorescence signal (at ca. 120 m). Seawater was filtered through Whatman GF/F filters (in triplicate) for the chlorophyll-a concentration determination.

### Analytical procedures

#### Benthic community

Meiofauna was extracted from the sediment by sieving through 500 µm and 45 µm mesh nets. The fraction retained was resuspended and processed according to the protocol reported by Danovaro [25]. All meiofaunal organisms were counted and classified to phylum or class level under a stereomicroscope. In order to obtain the functional parameter (biomass) of the meiofaunal component, the organisms were weighed after drying at 60°C for 24 h.

The macrofaunal specimens were recognised, when possible, down to species level. All the organisms, divided by species, were weighed to obtain the biomass value expressed as DW (drying at 60°C for 24 h). Molluscs and echinoderms were treated with 30% HCl prior to weighing.

#### Environmental variables

The grain size analysis was performed following Buchanan and Kain [26]; briefly, sediments were sieved (9 mesh sizes from 3.35 to 0.063 mm) after H_2_O_2_ treatment and drying (60°C, 48 h) and each fraction was weighed. Sediment particle-size diversity (Sed-H) was calculated from the percent dry weight of 5 size classes (<0.063 mm, 0.063–0.212 mm, 0.212–0.5 mm, 0.5–2 mm and >2 mm) using the Shannon–Wiener diversity index [27].

The chlorophyll-a concentrations in the water column were measured on board following the method of Holm-Hansen et al. [28], using a Perkin Elmer LS50B spectrofluorometer calibrated with chlorophyll-a from spinach (Sigma C5753). The specific standard deviation of the replicates was on average 4%.

The protein content of the sediment was determined following Hartree [29], the carbohydrate content was determined following Dubois et al. [30] and the lipid content was determined following Bligh and Dyer [31] and Marsh and Weinstein [32]. A Jasco V-500 spectrophotometer was calibrated with bovine serum albumin, glucose and tripalmitine solutions, respectively. Labile organic phosphorus was determined following the first step of the sequential extraction (SEDEX) proposed by Ruttenberg [33]. Briefly, sediment samples (3 to 5 g) were shaken for 2 h at 50°C in a 1 M MgCl_2_ solution in order to detach the loosely absorbed P from the sediment. The supernatant was then treated with an oxidising solution (K_2_S_2_O_8_) [34] in order to transform all the P into inorganic phosphates, which were then detected following Hansen and Grasshoff [35]with a SYSTEA Nutrient Probe Analyser. Organic carbon and total nitrogen were determined following Hedges and Stern [36] with a Carlo Erba Mod. 1110 CHN Elemental Analyser after acidification with hydrochloric acid to remove the inorganic carbonate fraction. Cyclohexanone-2,4-dinitrophenyl hydrazone was chosen as standard.

The hydrolytic enzymatic activities (β-glucosidase – BG, alkaline phosphatase – AP and leucine aminopeptidase – LA) were determined following Hoppe [37], using artificial substrates: 4-methylumbelliferyl β-D glucopyranoside and 4-methylumbelliferyl phosphate (excitation at 365 nm and emission at 460 nm) for BG and AP, respectively, and L-leucine 7-amido-4-methylcoumarin hydrochloride (excitation at 380 nm and emission at 440 nm) for LA. The samples and controls (sample sediment boiled as a blank for accidental contamination due to handling and for abiotic cleavage of the artificial substrates) were incubated in duplicate with 0.5 ml of substrate solution for 3 h. Incubations in the dark respected in-situ temperatures. Fluorescence was measured with a Perkin-Elmer 50 L spectrofluorometer previously calibrated with 4-methylumbelliferone and 7-amino-4-methylcoumarin solutions. The LA and BG activities were converted into equivalents of mobilised C assuming that 1 nmol of substrate hydrolysed enzymatically corresponded to 72 ng of mobilised C [38].

The two degradative enzymes were associated to their respective OM component: the LA with proteins, because the enzymatic hydrolysis of polypeptides is the preliminary step to amino-acid mineralisation [39], and the BG with carbohydrates, because the enzyme is involved in cellulose degradation [40]. AP activity has generally been associated with remineralisation of dissolved inorganic P, but it has bi-functional features [41]. We related it to labile P. The OM turnover times were calculated by converting the proteins and carbohydrates into C equivalents (factors of 0.49 and 0.40 for proteins and carbohydrates, respectively, according to the C content of the standard) and then dividing by the LA and BG activities transformed into their equivalents of mobilised C. The labile phosphorus turnover time was calculated following the same procedure, but assuming that 1 nmol of substrate hydrolysed enzymatically by AP corresponded to 31 ng of potentially released phosphate [38].

### Statistics

We tested the differences in the same variable between different samplings with the one-way ANOVA test followed by the Newman-Kneuls post-hoc test (ANOVA+NK test) (Statistica software). To test the relationships between the various parameters, a Spearman-rank correlation analysis was performed. We used the PRIMER 6β programme package to perform SIMPER analysis on the metazoan abundance data, separately for meiofauna and macrofauna. The data matrices have been transformed using presence/absence. DISTML (distance-based linear model) routines were performed with the PERMANOVA+ programme package for PRIMER to analyse and model the relationship between the meiofaunal abundance and the environmental variables. Of the original set of environmental parameters, 9 were retained for further analysis. The variables with correlation R^2^ values >0.9 (considered redundant)were omitted for the DISTLM procedures. The meiofaunal DISTML was constructed using the step-wise selection procedure and the adjusted R^2^ as selection criterion to enable the fitting of the best explanatory environmental variables in the model. Euclidean distance was used as resemblance measure.

A PCA analysis, based on to the trophic quality of the OM, was performed of the 4th-root-transformed data (reported in Table 2) to reveal similarities between stations.

**Table 2:** Quality indexes of the OM: C/N ratio, protein/carbohydrate ratio, turnover times (days) in the two sediment layers.

station	area	C/N ratio		protein/carbohydrate ratio		carbohydrate turnover		protein turnover		labile P turnover	
		0–2 cm	2–10 cm	0–2 cm	2–10 cm	0–2 cm	2–10 cm	0–2 cm	2–10 cm	0–2 cm	2–10 cm
53	far-field	14.8±0.8	13.5±1.6	0.23	0.15	379	823	2.6	1.6	0.09	0.19
25	deeper flank	20.7±2.8	10.6±0.1	0.34	0.24	218	840	1.9	3.2	0.04	0.25
9	medium flank	19.7±2.7	15.2±0.4	0.11	0.30	549	653	1.5	3.9	0.02	0.10
28	upper flank	12.4±1.1	15.0±2.9	0.16	0.32	382	768	0.8	2.1	nd	0.09
14	upper flank	11.7±0.9	10.5±1.8	0.11	0.10	179	997	3.7	3.0	0.01	0.06
16	medium flank	11.8±0.7	11.0±1.0	0.14	0.20	223	264	1.2	1.6	0.03	0.07
32	deeper flank	13.6±0.8	13.6±0.2	0.20	0.24	826	1332	1.8	2.4	0.08	0.18
41	far-field	12.0±0.8	12.6±0.3	0.43	0.27	427	854	7.6	8.2	0.09	0.11

## Results

### Benthic communities

**Figure 2:**
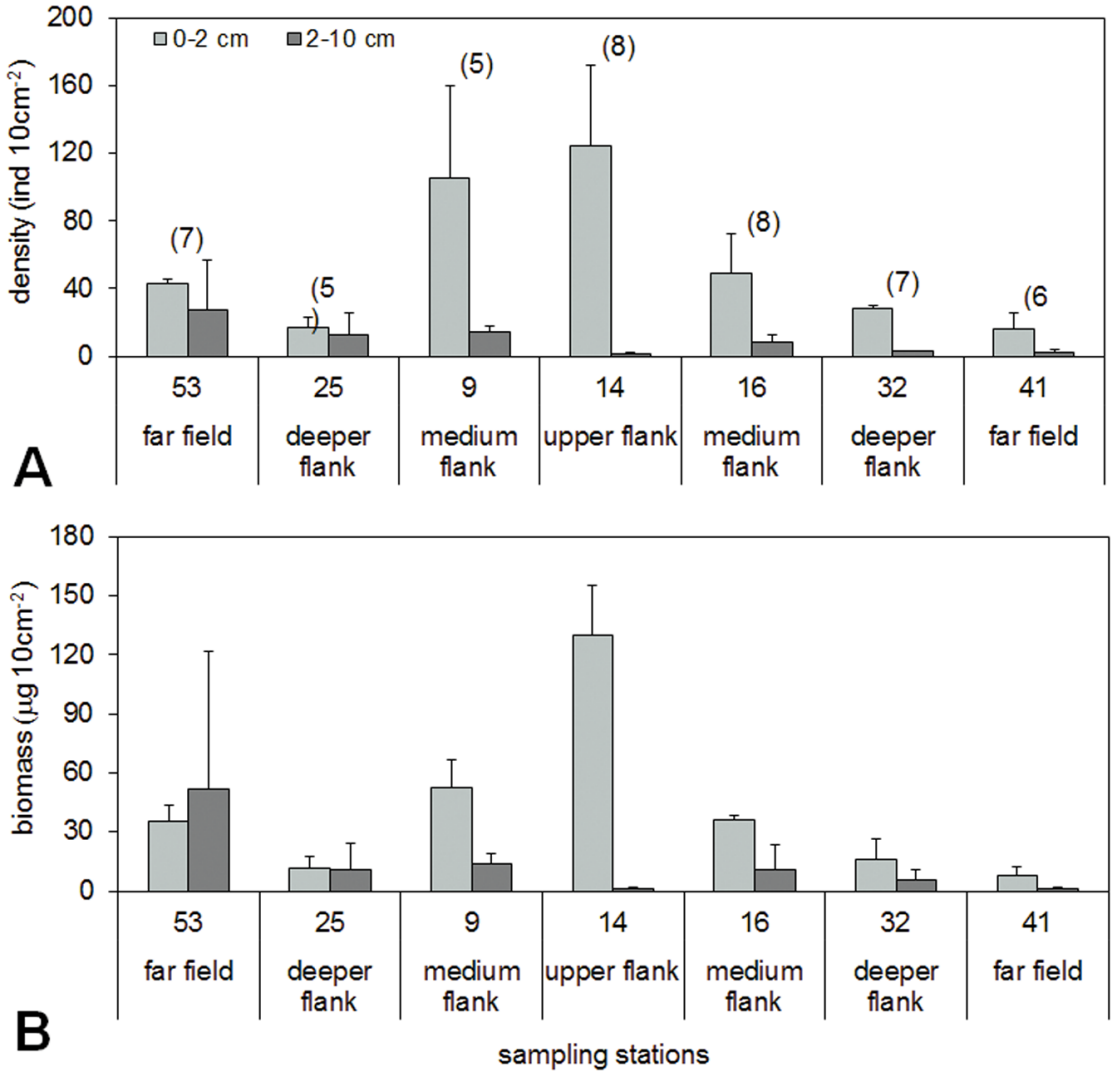
Preliminary meiofaunal abundance and biomass data for the two sediment layers of each station. Values averaged over replicates collected in the same deployment: bars denote standard error. A: abundance (individuals 10 cm^−2^), in brackets the number of taxa, B: biomass (µg 10 cm^−2^, the values have been calculated with the gravimetric method).

The meiofaunal communities had their highest densities at the stations situated on the upper and medium flanks of the seamount (Fig. 2A). The total abundance, although it has to be considered as preliminary data, decreased significantly with water depth (R = −0.82, n = 7, p<0.05), a trend confirmed also by the DISTLM analysis (Table 3). The first 2 cm depth was the layer mainly inhabited by the organisms (85–99% of the total abundance) at most of the stations, although in the northwestern area (stations 53 and 25) the vertical distribution was more homogeneous: only 62% and 58%, respectively, preferred the surface sediments (Fig. 2A). Overall 13 taxa were found in the study area, 11 at the seamount stations (Table 4), although the limited sampling procedure could have not highlighted all the rare species in the different sites. The number of taxa ranged between 5 (stations 9 and 25) and 8 (stations 14 and 16, Fig. 2A). The assemblage abundances were dominated by nematodes and copepods with nauplii (61–76% for nematodes and 5–11% for copepods). The community structure was similar in the different parts of the seamount and far field (SIMPER: dissimilarities from 33.6% to 23.8%). The biomass patterns were similar to the abundance ones (Fig. 2B), except at station 53, which had a higher biomass in the deeper sediments due to the high number of polychaetes found. The higher biomass contributions were given by nematodes (26–71%) and polychaetes (0–69%), while copepods represented only 4–9%.

**Table 3:** Distance-based linear model (DISTLM) for meiofaunal abundance and selected environmental variables.

MARGINAL TESTS					
Variable	SS(trace)	Pseudo-F	P	Prop.	
H sed	2323.2	3.788	0.052	0.43	
water depth	3688.4	10.840	0.016	0.68	
protein turnover time	418.6	0.421	0.597	0.08	
carbohydrate turnover time	471.5	0.479	0.542	0.09	
labile P turnover time	2295.3	3.709	0.107	0.43	
proteins	1468.1	1.872	0.240	0.27	
carbohydrates	4276.6	19.212	0.002	0.79	
lipid	469.6	0.477	0.510	0.09	
labile P	1154.5	1.363	0.277	0.21	
SEQUENTIAL TESTS					
Variable	Adjust.R^2^	SS(trace)	Pseudo-F	P	Prop.
+carbohydrates	0.7522	4276.6	19.212	0.003	0.79
+labile P	0.7561	236.6	1.080	0.364	0.04
+water depth	0.8380	439.8	3.022	0.140	0.08
+carbohydrate turnover time	0.9452	338.2	6.870	0.060	0.06
+protein turnover time	0.9857	85.6	6.660	0.172	0.02

Marginal tests: explanation of variation for each variable taken alone. Sequential tests: conditional tests of individual variables in constructing the model. Each test examines whether adding the variable contributes significantly to the explained variation. Selection procedure: step-wise, selection criterion: adjusted R^2^. Prop.: % variation explained.

**Table 4:** List of the meiofaunal taxa found in the sampling stations and position of each station with respect to the seamount morphology.

stations taxa	53 far-field	25 deeper flank	9 medium flank	14 upper flank	16 medium flank	32 deeper flank	41 far-field
Amphipoda						1.3	
Copepoda	6.1	10.0	8.2	9.2	4.2	11.3	7.4
Nauplii		2.5	0.4	1.7	1.2		
Gastrotricha					0,6		
Kinorhyncha	0.5						
Nematoda	79.7	81.2	82.6	78.1	85.5	77.5	83.3
Oligochaeta					1.2		
Ostracoda	1.0		1.7	0.6	0.6		1.8
Polychaeta	11.7	3.8	3.2	8.3	4.2	3.7	
Rotifera	0.5		3.9	1.2	1.9	2.5	1.9
Sipuncula		1.3		0.3			
Tanaidacea				0.3			
Thermosbaenacea	0.5			0.3		2.5	1.9
Turbellaria		1.3			0.6	1.2	3.7

Percentage contributions of the taxa to the meiofaunal total abundance for each station are presented.

**Figure 3:**
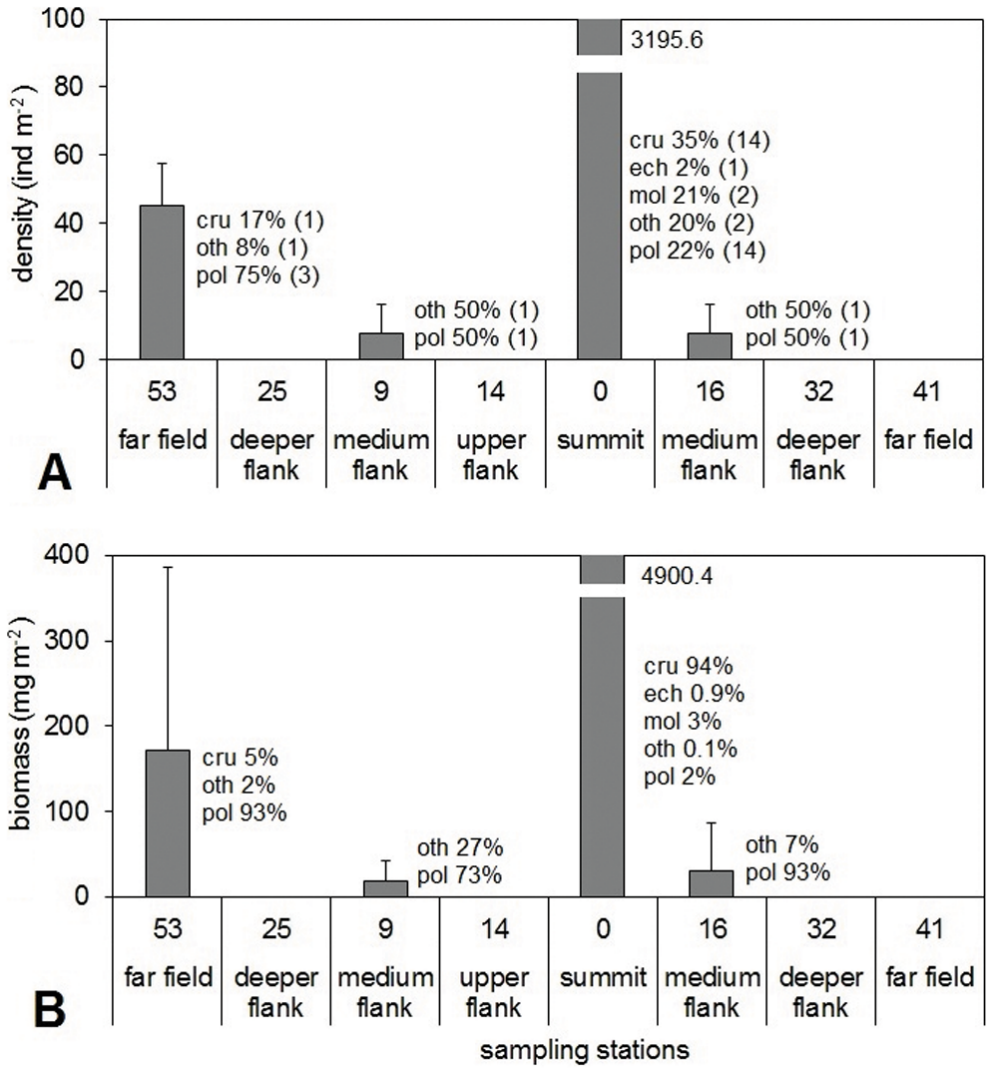
Average macrofaunal abundance and biomass and the contribution of each taxonomical group (cru: crustacean, pol: polychaetes, mol: molluscs, ech: echinoderms and oth: others) for each station. Values averaged over independent replicates: bars denote standard error. A: abundance (individuals m^−2^), in brackets the number of taxa, B: biomass(µg m^−2^, the values have been calculated with the gravimetric method).

Macrofauna was only found at four stations (53, 9, 16 and 0, Fig. 3A), characterised by depths shallower than 1500 m. It seems that the presence of macrofauna was related to depth, but actually station 14 (390 m depth) showed no macrofaunal organisms, indicating that features other than depth must be involved. Macrofaunal densities were lower than 50 ind m^−2^ at stations 53, 9 and 16, but at station 0 the value was greater than 3000 ind m^−2^. The number of taxa was also low at stations 53, 9 and 16, with four species of polychaetes, one species of crustacean, and two species of sipunculans. Station 0, instead, showed a high diversification (Table 5) and was dominated by crustaceans. Molluscs, polychaetes and others showed similar contributions; only echinoderms were scarce (Fig. 3A). The SIMPER analysis showed higher dissimilarities between assemblages of the seamount flanks and of the far field area (85.7%), between the assemblages of the seamount flanks and of the summit (100%) and between the assemblages of the far field and of the summit (94.7%). The macrofaunal biomass showed patterns similar to the density, but the taxa contribution was different: at the summit station crustaceans accounted for 94%, while at the other stations polychaetes made higher contributions (Fig. 3B).

**Table 5:** List of the macrofaunal taxa found on the Vercelli Seamount summit (station 0), medium flank (stations 9 and 16) and in the far-field area (station 53).

Phylum	Class	Family	Species	stations		
				0	9–16	53
**Annelida**	**Polychaeta**					
	Scolecida	Capitellidae sp1			X	
		Capitellidae sp2		X		
		Opheliidae		X		
		Paraonidae sp1				X
		Paraonidae sp2		X		
	Aciculata	Chrysopetalidae sp1		X		
		Chrysopetalidae sp2		X		
		Exogoninaejuv.		X		
		Goniadidae		X		
		Hesionidae		X		
		Nereididae		X		
		Phyllodocidae sp1				X
		Phyllodocidae sp2		X		
		Sigalionidae		X		
		Syllidae sp1		X		X
		Syllidae sp2		X		
**Artropoda**	**Malacostraca**					
	Decapoda	Leucosiidae	*Ebalia* sp	X		
		Parthenopidae	*Parthenopoides massena*	X		
	Amphipoda	Ampithoidae	*Ampithoe helleri*	X		
		Leucothoidae	*Leucothoe occulta*	X		
		Lyssianassidae		X		
	Tanaidacea	Apseudidae	*Apseudopsis elisae*	X		
			*Apseudes* sp			X
	Isopoda sp1			X		
	Isopoda sp2			X		
	Isopoda sp3			X		
	Isopoda sp4			X		
	**Maxillopoda**					
	Copepoda			X		
	**Ostracoda**	Cypridinidae		X		
		Bythocytheridae		X		
**Mollusca**	**Bivalvia**	Limidae	*Limaria hians*	X		
		Lucinidae	*Lucinella divaricata*	X		
**Echinodermata**	**Ophiuroidea**	Amphiuridae	*Acrocnida brachiata*	X		
**Sipuncula**	**Sipunculidea**	Phascolionidae	*Phascolion (Phascolion) strombus*		X	
		Phascolosomatidae	*Phascolosoma (Phascolosoma) granulatum*		X	X
**Platyhelminthes**	**Turbellaria**			X		
**Nematoda**				X		

### Environmental variables

#### Seawater autotrophic biomass

**Figure 4:**
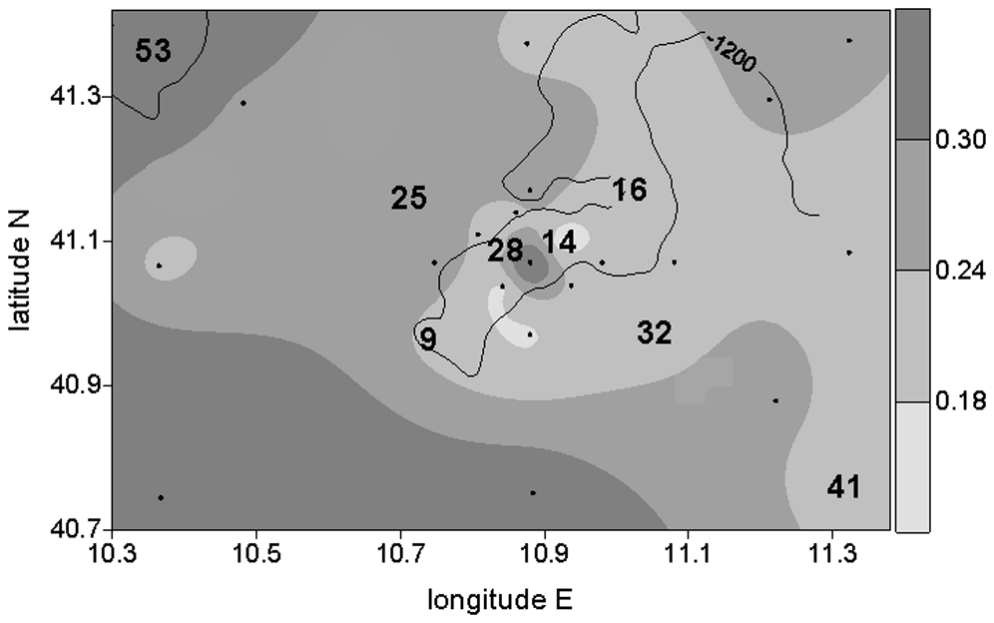
Seawater autotrophic pigment distribution. Chlorophyll-a concentrations (µg l^−1^) in the water column have been averaged for the 0–120 m layer. The numbers indicate the stations also sampled for the sedimentary parameters, the dots indicate all the stations sampled for the autotrophic biomass determination in the water column. The line reports the 1200 m depth isobath.

The distribution of the autotrophic biomass in the water column (integrated values in the 0–120 m layer for the chlorophyll-a concentration) is shown in Fig. 4. The highest values in the area studied were found at station 0 (seamount summit, 0.43 µg l^−1^) and in the SW and NW sectors (0.41 µg l^−1^ at station 53). The waters surrounding the summit had low (0.14 µg l^−1^ at station 14) and rather low (from 0.22 to 0.24 µg l^−1^ at stations 9, 16, 28 and 32) values. The SE sector also displayed rather low values (0.20 µg l^−1^ at station 41) while values higher than 0.24 µg l^−1^ were observed in the NE sector.

#### Sediment texture

**Figure 5:**
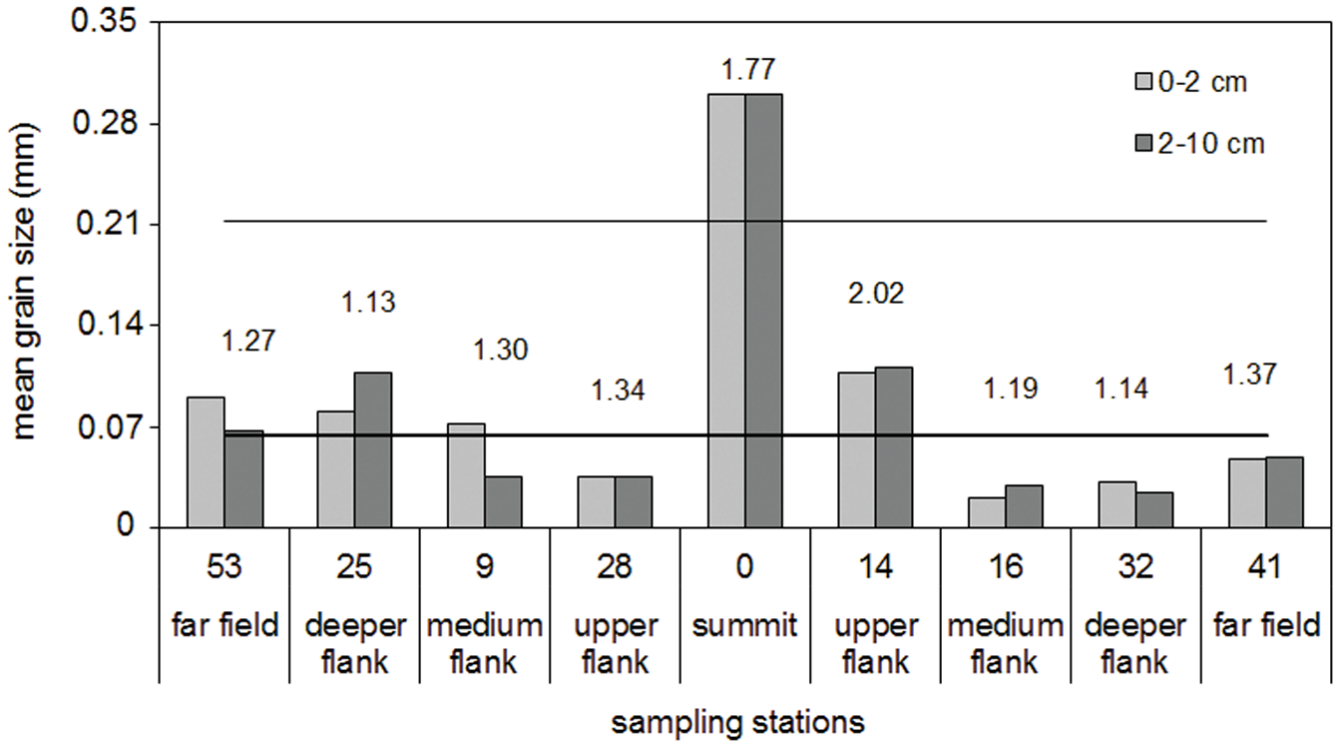
Sediment texture. Mean grain size (mm) for the sediment 0–2 cm layer and 2–10 cm layer. The black horizontal line highlights the upper limit of the silt & clay fraction (0.064 mm), the grey line the division between fine and medium sand (0.212 mm). The numbers above the bars report the sedimentary diversity index (Sed-H) for each station.

The mean grain size of the sediment (Fig. 5) was in the range of silt & clay for half of the sampled stations, while only the shallowest station 0 had a medium-sand texture. The coarser grain sizes (gravel, >2 mm) were found only at the shallowest stations 0 and 14, while the silt & clay fraction was highly represented in the 0–2 cm layer of station 16 (40%). On average, the most represented fraction was that ranging from 0.064 to 0.212 mm (52%), followed by the fraction ranging from 0.212 to 0.5 mm (30%) and by the fraction lower than 0.064 mm (15%).

The Sed-H results (Fig. 5) highlighted the highest sediment diversity at stations 0 and 14, and the lowest at stations 25 and 32. The sediment diversity was significantly correlated to the meiofaunal biomass (R = 0.77, n = 7, p<0.05), and also the meiofaunal density was directly proportional to the sediment diversity, although not significantly (Table 3).

#### Sedimentary organic matter (OM)

**Figure 6:**
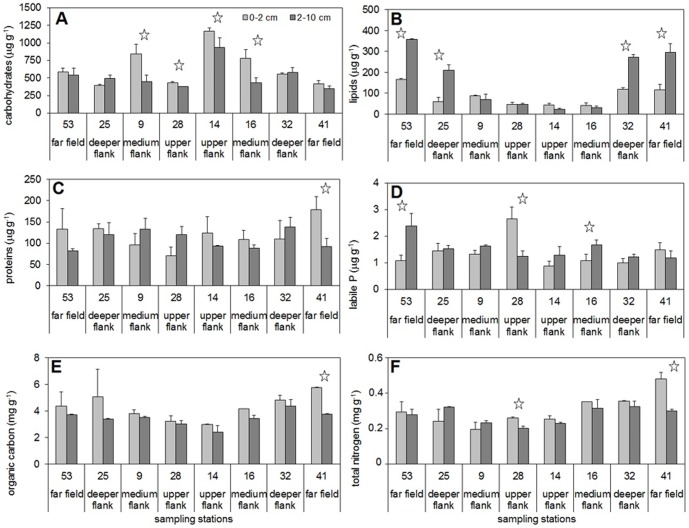
Organic matter contents for the two sediment layers. Bars denote standard deviations. A: carbohydrates (µg g^−1^), B: lipids (µg g^−1^), C: proteins (µg g^−1^), D: labile P (µg g^−1^), E: organic carbon (OC, mg g^−1^), F: total nitrogen (N, mg g^−1^). Stars denote significantly different values for the 0–2 cm and the 2–10 cm sediment layers at each station (one-way ANOVA, NK post-hoc test, p<0.05).

The carbohydrate content (Fig. 6A) had the highest values of the OM components (on average 583.1±232.2 µg g^−1^ for the two layers). All the upper- and medium-flank stations of the seamount showed 0–2 cm layer values significantly higher than the 2–10 cm layer ones (one-way ANOVA and NK post-hoc test, p<0.05), with stations 9, 14 and 16 reaching the highest absolute values (from 776.9±125.6 to 1169.3±43.4 µg g^−1^ for stations 16 and 14, respectively). Considering the 0–2 cm layer, the correlation between meiofauna abundance and carbohydrates was highly significant (R = 0.95, n = 7, p<0.01). The DISTLM analysis confirmed that, within the environmental trophic features, the carbohydrate content was the variable mainly linked to the meiofaunal abundance.

Lower values were observed for the lipid content (on average123.7±106.2 µg g^−1^for the two layers), even lower if the upper- and medium-flank stations of the seamount were considered (on average 47.9±21.0 µg g^−1^) (Fig. 6B). This difference was significant (one-way ANOVA and NK post-hoc test, p<0.01). On the other hand, no significant differences were observed between the two sediment layers of the upper-and medium-flank stations of the seamount, while the other stations showed a significant accumulation in the 2–10 cm layer (one-way ANOVA and NK post-hoc test, p<0.01).

The protein content values (Fig. 6C) were more homogeneous throughout area (on average 114.0±27.1 µg g^−1^), without significant differences except for the two layers of station 41 (one-way ANOVA and NK post-hoc test, p<0.05). The labile P values (Fig. 6D) did not show significant differences between the stations. The OC and N contents (Figs. 6E and 6F) of the two layers of the upper- and medium-flank stations of the seamount, considered together, showed significantly lower values than the other stations (one-way ANOVA and NK post-hoc test, p<0.01). Significant differences between the two layers were rare for protein, P labile, OC and N contents.

#### Sedimentary enzymatic activity and OM turnover

**Figure 7:**
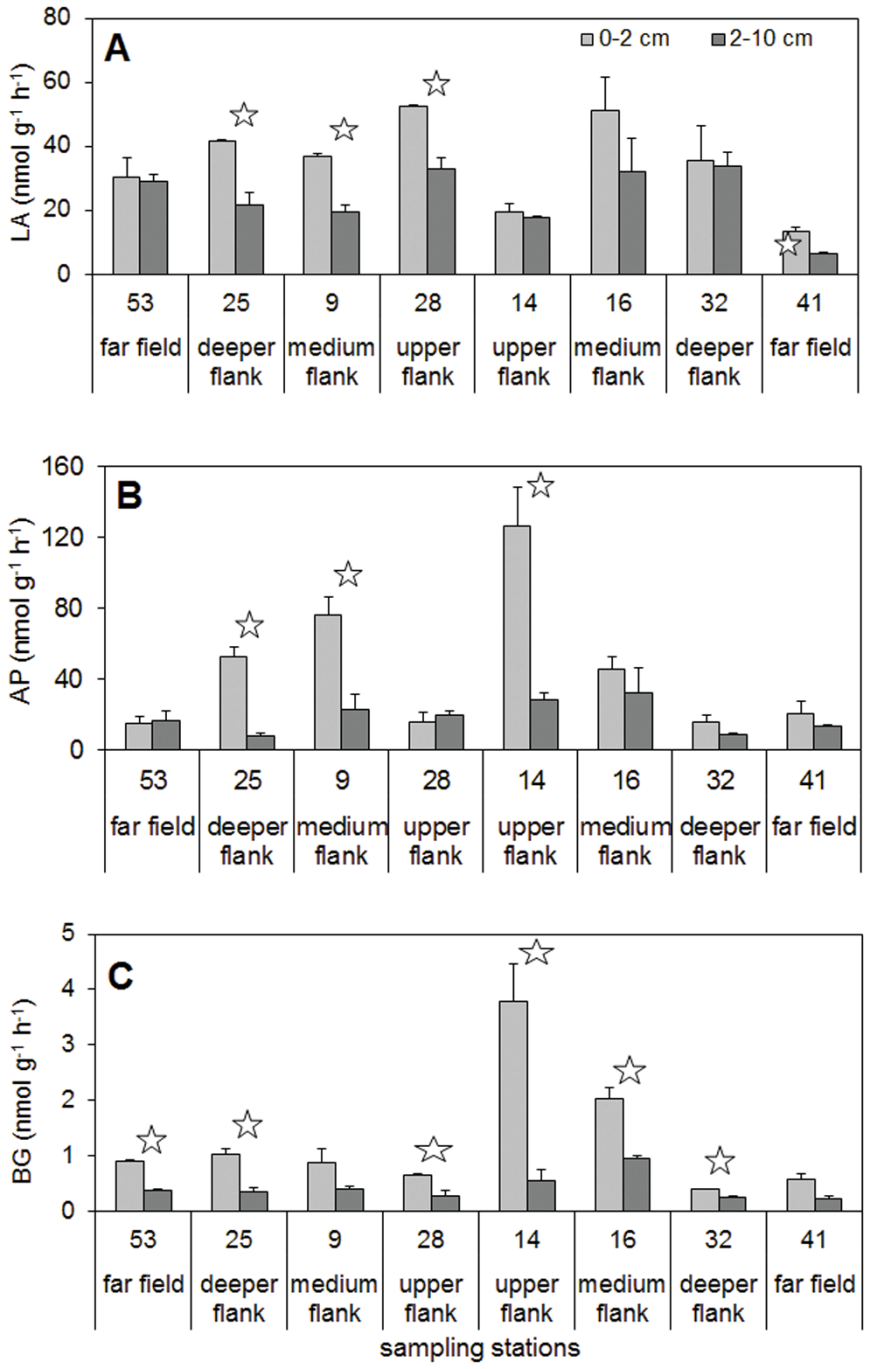
Enzymatic activities (nmol g^−1^ h^−1^) for the two sediment layers. Bars denote standard deviations. A: leucine aminopeptidase (LA), B: alkaline phosphatase (AP), C: β-glucosidase (BG). Stars denote significantly different values for the 0–2 cm and the 2–10 cm sediment layers at each station (one-way ANOVA, NK post-hoc test, p<0.05).

The enzymatic activities are shown in Fig. 7 and the related OM turnover times in Table 2.

The LA activity was on average 29.7±12.8 nmol g^−1^h^−1^ for the whole area and the stations didn't show significant differences. The surface sediment layer always had higher values than the deep one, sometimes significant (one-way ANOVA and NK post-hoc test, p<0.05). The protein turnover times were, on average, 2.9±2.1 days for the whole area and the surface layer showed generally lower values than the deep one, except for stations 53 and 14. The deepest station, station 41, showed the highest value, followed by the shallowest, station 14.

The AP activity showed the highest values in the 0–2 cm layer of station 14, but station 9 also showed notable activity in its surface layer. The turnover times were significantly (one-way ANOVA and NK post-hoc test, p<0.05) lower for the upper- and medium-flank stations than for the other stations (on average 0.05±0.03 vs 0.13±0.07 days, respectively).

The BG activity was higher at stations 14 and 16, lower at the southernmost stations 32 and 41, and rather high variability was recorded for the upper- and medium-flank stations. The surface sediment layer showed significantly higher BG values than the deep layer (one-way ANOVA and NK post-hoc test, p<0.05). The carbohydrate turnover times were very high (on average 607±332 days) and the surface layer showed lower values than the deep one.

**Figure 8:**
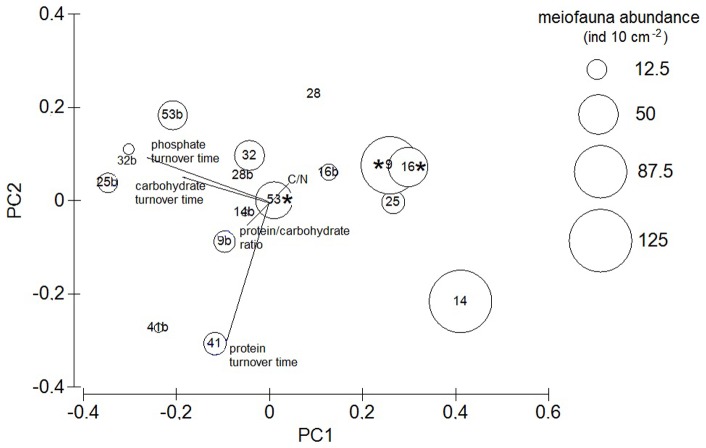
PCA analysis of the OM trophic-quality features (see text and Table 2 for details) for the upper flank stations 14 and 28 (at 400 and 877 m depth), the medium-flank stations 9 and 16 (at 1232 and 1166 m depth), the deeper-flank stations 25 and 32 (at 1833 and 1728 m depth), the far-field stations 53 and 41 (at 887 and 2646 m depth). C/N: (carbon/nitrogen ratio) the lower the ratio the higher the trophic quality, protein/carbohydrate: the higher the ratio the higher the trophic quality, protein-carbohydrate- labile P turnover times: the lower the time the higher the trophic quality. Meiofaunal total biomass is superimposed as bubbles and asterisks denote where macrofauna was found. “b” after the station number denotes the 2–10 cm layer of the station.

Keeping in mind that the meiofaunal data were preliminary due to the limited sampling procedure, the DISTML analysis (Table 3) indicated that the best model for the meiofaunal abundance and the environmental features included, together with the water depth and the carbohydrate sedimentary content, the turnover times for proteins and carbohydrates. The PCA analysis (Fig. 8), focused on the indexes of OM lability, showed thatPCA1 and PCA2 explained 78.5% of the variation. The superimposed bubbles are proportional to the meiofaunal abundances and the asterisks indicate where macrofauna were found.

The statistical analyses indicated that the C/N and the protein/carbohydrate ratios failed to give a reasonable picture of the OM lability, at least for the sediments. For instance, the highest protein turnover times of station 41, indicating the poor trophic quality of the protein content, disagrees with the high protein/carbohydrate ratio values, thus indicating refractory protein accumulation rather than a large pool of trophic resources. This may be related to an analytical bias, because the protein method overestimates the concentration of proteins because of the presence of humic substances [42].

## Discussion

The Tyrrhenian basin has been considered as one of the more oligotrophic of the western Mediterranean in terms of water column features [43]. In agreement with this scenario, previous studies on meiofauna [8] highlighted rather low abundances compared to extra-Mediterranean ones (in the Atlantic Ocean, for instance) [16], while benthic prokaryote abundance and biomass fell within the range of values reported in the literature for deep-sea sediments worldwide [7]. However, no exhaustive information has been provided for the deep Tyrrhenian Sea macrofaunal organisms, nor for the seamount-summit macrofauna of this area.

The summit station, 0, is a world apart for macrofauna, due firstly to the shallow depth (within the photic zone and enriched by good trophic-quality phytopigments) and to a coarse granulometry that allows for deep oxygenation of the sediment. The heterogeneity of the substrata at the summit (rocky substrate alternating with coarse, biogenic debris) may provide different habitats for a high number of species [15]. In addition, the presence of a *Laminaria rodriguezii* meadow at the seamount summit [11]would provide the macrofauna with a surplus of food resources through the detrital food chain. In fact, as reported for the coastal *Posidonia oceanica* meadows of the Ligurian Sea [44], the macrobenthic community at the summit is dominated by carnivores (94% of the total abundance) such as Aciculata families (89% of polychaetes), Decapoda, Turbellaria, Lyssianassidae and the amphipod *Leucothoe occulta* (Krapp & Schickel, 1975) (Table 5). The good quality of the organic particulate matter [45] favours suspension feeders and mixed suspension-deposit feeders (ca. 6%) such as the echinoderm *Acrocnida brachiata* (Montagu, 1804), the bivalve *Lucinella divaricata* (Linnaeus, 1758) and cypridinid ostracods (Table 5). On the contrary, only deposit and mixed suspended-deposit feeders, such as Scolecida and Sipuncula, were found at the medium-flank stations (Table 5).

In our study we observed that, with the exception of the summit station, the benthic communities of the Vercelli Seamount area were poor in macrofauna (for instance, lower than at the Atlantic Condor Seamount [15]) and moderately rich in meiofauna (abundance and number of taxa higher than those reported for the sediments surrounding the Tyrrhenian Palinuro and Marsili seamounts[8] but lower than the abundances found at the Atlantic Condor Seamount [16]). Benthic communities are generally influenced by water depth [16], [46]. In accordance with the above, a significant correlation between depth and our preliminary data of total meiofaunal abundance was found, and the macrofaunal organisms have only been found at some shallower stations.

Leduc et al. [47] showed that the sediment texture, transformed into a sediment-diversity index (Sed-H) by means of the Shannon-Wiener diversity index, deeply influences soft-bottom communities, especially those groups, such as meiofauna, that have a limited expansion rate due to their small size. In our study the preliminary data on meiofaunal abundance agreed with these observations, showing the highest abundances and biomasses where the Sed-H was the highest (station 14) and the lowest where the Sed-H was the lowest (stations 25 and 32).

Macrofauna did not directly respond to the Sed-H as the meiofauna did. Considering other environmental features that may be involved in the macrofauna distribution, we observed that three stations (9, 16 and 28, although the latter was not sampled for benthic communities) had a particular exposition degree, opposite that of the regular seamount flank slope. This implies the presence of irregularities along the flank that interfere with the incoming current, modifying the local hydrodynamic processes, allowing slowing of the current speed and increasing deposition. This would explain the fine granulometry of these stations, finer than the other flank-stations. Higher sedimentation rates on the northern flank of the seamount were also revealed by the composition and trophic strategies of the megafauna assemblages found there by Bo et al. [11]. The peculiar environment generated by these morphological features seems to favour macrofaunal development. Except for the summit station, only stations 9 and 16 within the very poor area of the Vercelli Seamount showed the presence of macrofaunal organisms. The lack of macrofauna at shallow station 14 may, then, be related to the high bottom slope and to an excessive hydrodynamic forcing, as this station lacks appropriate aprons. In addition, the high meiofaunal density and biomass at this site may have exerted more efficient competition with macrofauna and/or a top-down control of macrofaunal juveniles [48].

However, water depth, sediment texture and sediment aspect are not enough to explain benthic community distribution [17], [19], especially in a variable environment such as the Vercelli Seamount area. The quantitative features of the trophic supply in deep sediments have been indicated as a limiting factor for the growth and activity of microbes [39] and small metazoan [49], and their qualitative features (bioavailability) have been invoked to explain community distribution as well [1], [50].

Surprisingly, but in agreement with previous literature data [8], our preliminary data on meiofaunal abundance were related to a rather refractory fraction of the organic matter (OM), namely the carbohydrates, indicating that in this peculiar system the carbohydrate trophic supply would play a role in the distribution of the meiofaunal organisms. The absence of significant relationships for the 2–10 cm layer suggested that these carbohydrates may derive from deposition via pelagic-benthic coupling.

In fact, the shallower seamount stations (9, 28, 14 and 16) showed a significant enrichment of the upper sediment layer, indicating that this carbohydrate distribution was related to the flux of OM from the surface layer. Frontal zones, as observed in the Vercelli Seamount area, are known to induce the sinking of particulate material towards the deeper layers [39]. Therefore, most of the organic bulk in these benthic areas is composed of high-molecular-weight compounds, so that the benthic zone is enriched with surface OM, which usually does not reach the seafloor. The phytoplanktonic debris, the main OM source in pelagic environments, was probably highly involved in these processes, as suggested by Speicher et al. [51]. While the more labile components of the OM flux (proteins and lipids, for instance) could be consumed and re-cycled during their sinking to the sediment; structural carbohydrates (cellulose, for instance) were preserved and reached the sediments at a higher rate.

The complex hydrodynamic assessment of the studied area led to a carbohydrate accumulation that did not exactly match the chlorophyll-a distribution in the water column recorded during the same sampling cruise. The pelagic-benthic coupling was based on oblique fluxes rather than on vertical ones; the fluxes (and the accumulation) being higher in the shallower stations where the dilution is proportionally lower. In the southernmost section of the sampling area, the Bonifacio gyre flows from the SW [13], reaching the seamount area at station 9. The favourable conditions to phytoplanktonic biomass accumulation in the surface layer of the SW sector, that led to the very high chlorophyll-a signal recorded, could have generated a high OM flux, carrying high carbohydrate amounts to the sediments of station 9. During our cruise high chlorophyll-a values were also measured in the water column of the NE section. Sinking phytoplanktonic biomass, pushed by the gyre previously reported by Vetrano et al. [13], could have reached our station 16, increasing the carbohydrate content of the surface sediments. Station 14 was probably the only one directly subjected to the “seamount effect”, indicated by the increase of phytoplanktonic biomass centred on the seamount summit that we recorded. Lateral transport of this OM could have reached shallow station 14, generating the significantly higher carbohydrate contents of the surface sediment.

Although the carbohydrate contents of the entire Vercelli Seamount area showed rather high values, the other biochemical fractions were lower than those reported for other Mediterranean and Atlantic areas [8], [52], studied using the same analytical methods. The C-OM (namely the sum of the C contribution of proteins, carbohydrates and lipids) of the Portuguese, Adriatic and Cretan margins was three times the average value we found in the Vercelli Seamount area. Our results confirmed the general oligotrophy of the Tyrrhenian Sea and the tendency to ultraoligotrophy of the Vercelli area, highlighted by a very low protein content [53].

The depositional features of the studied area may have a role in these low values. Speicher et al. [51] highlighted a rather low particulate organic carbon (POC) export flux in the northern Tyrrhenian Sea (where the Vercelli Seamount is placed) during late May-early June compared to the middle and southern Tyrrhenian. In addition, it is well known that the sediment texture regulates the OM accumulation, the coarser sediments providing a lower surface to be coated by OM. The generally low contribution of the silt & clay fraction (grain size <63 µm) in the Vercelli area (on average 15%) compared to the 22–98% of the slope areas reported by Pusceddu et al. [8] agrees with this statement.

Together with the common quality indexes (C/N ratio and protein/carbohydrate ratio), the hydrolytic enzymatic activity may give clues on the potential lability of the OM [41], following the optimal resource allocation model of Sinsabaugh et al. [54], who stated that osmotrophic assemblages will optimize their energy expenditures by expressing high levels of particular hydrolases only if polymeric substrates are abundant and if the monomeric hydrolysates required for growth are scarce. The low values of the substrate quantity: enzyme activity ratio, namely low turnover times, highlight high trophic value OM. A major role of the OM turnover times was observed, and the highest meiofaunal abundances were found in stations where the OM was more labile (surface layer of stations 9, 14, 16) as indicated by the multivariate analysis.

The BG activity and the carbohydrate turnover time may, therefore, be useful to explain meiofaunal as well as macrofaunal distribution. BG activity was always higher in the surface sediment layer, probably becoming too energy expensive for the trophically-limited deeper sediment layer. This matches the vertical meiofaunal distribution, with more than 80% of the total abundance found in the surface layer. Considering, instead, the macrofauna, its biomass value at station 16, higher than that of station 9, was in agreement with the higher trophic quality of the sediment, characterised by rather low turnover times for carbohydrates and proteins. In fact station 9, although showing a notable carbohydrate content, showed higher turnover times, indicating a lower lability for the OM reaching the bottom, due probably to the longer time the OM spent in the water column.

The deepest station, station 41, showed the highest protein turnover time values in both layers, indicating that the accumulated proteins did not induce LA activity as they did at the other stations of our study area. This may be because peptidase activity has been described as being negatively correlated to depth [55] but, from a trophic point of view, it indicates the lower lability of station 41 protein content. At the seamount-influenced sites, although the fluxes and/or in situ production were low, the proteins were generally more labile than those of the non-seamount station 41.

The relationship between benthic organisms and OM lability, expressed as a short turnover time, is complex. In fact, macrofaunal presence and also the preliminary information on meiofaunal abundance may explain the anomalous turnover times for proteins we detected for stations 53 and 14, which showed higher values in the surface sediment than in the deeper layer. If the coarser grain size of these stations allowed for higher mixing of the OM of the surface and deep sediment layers, station 53 showed high meiofaunal and rather high macrofaunal abundances, and the diffusion of OM is strongly stimulated by macrofaunal galleries [56]. At station 14, instead, the bioturbation was limited to the action of meiofauna, which showed the highest densities, due to the lack of macrofaunal organisms.

## Conclusions

The Mediterranean Sea has been studied intensively, but its deep areas need more research to increase our understanding of its ecosystem functions. In particular, the studies on the deep areas of the Tyrrhenian Sea are scarce, although its morphological features (seamounts, for instance) suggest the presence of diversified habitats and ecosystems. In the present paper we observed that in the Vercelli Seamount area the peculiar environmental features (physical, morphological and trophic) differently shaped the benthic assemblages. Macrofauna showed different community composition comparing the seamount flanks and the far-field stations, and the summit station was a world apart in terms of density, diversity and biomass. Bottom inclination and aspect allowed the presence of aprons against the hydrodynamic forcing, favourable to macrofauna development. Our preliminary data on meiofaunal abundance showed, instead, a link to water depth and to trophic supply, especially to the lability of the OM. A variable pelagic-benthic coupling links the seawater with the bottom indicating that, irrespectively of being far from the coast and/or placed in the deep sea, these areas may be sensitive to global processes.
